# Nucleot(s)ide Analogues for Hepatitis B Virus-Related Hepatocellular Carcinoma after Curative Treatment: A Systematic Review and Meta-Analysis

**DOI:** 10.1371/journal.pone.0102761

**Published:** 2014-07-24

**Authors:** Ping Sun, Xiaochuan Dong, Xiang Cheng, Qinggang Hu, Qichang Zheng

**Affiliations:** 1 Hepatobiliary Surgery Centre, Union hospital, Tongji Medical College, Huazhong University of Science and Technology, Wuhan, China; 2 Department of Pathology, Union hospital, Tongji Medical College, Huazhong University of Science and Technology, Wuhan, China; Osaka University Graduate School of Medicine, Japan

## Abstract

**Aim:**

The benefit of nucleot(s)ide analogues (NA) for hepatitis B virus (HBV)-related hepatocellular carcinoma (HCC) after curative treatment has been widely debated due to the relatively weak evidence. The objective of this systematic review was to evaluate the effect of NA on recurrence and survival after curative treatment of HBV-HCC.

**Methods:**

A systematic electronic search was performed. All controlled trials comparing NA versus placebo or no treatment were considered for inclusion. Results were expressed as Hazard Ratio for recurrence and survival with 95% confidence intervals using RevMan 5.2.

**Results:**

We included 13 trials with 6350 patients. There were significant improvements for recurrence-free survival (HR 0.66, 95% CI 0.54–0.80; p<0.0001) and overall survival (HR 0.56, 95% CI 0.43–0.73; p<0.0001) in the adjuvant NA group compared with the control group. Sensitivity analyses confirmed the robustness of the results. There were no serious adverse effects being reported. Lamivudine resistance was from 28.6% to 37.5% but could be rescued by other types of NA or combination therapy.

**Conclusion:**

Our study suggested benefits of adjuvant NA therapy following curative treatment of HBV-HCC. Since the great proven efficacy of NA in improving clinical and viral parameters besides HCC, further studies should be focused on broadening the indications for NA therapy after curative treatment of HBV-HCC.

## Introduction

Liver cancer is one of the most common cancers diagnosed worldwide [1]. Among primary liver cancers, hepatocellular carcinoma (HCC) represents the major histological subtype, accounting for 70% to 85% of the total liver cancer burden [2]. Hepatitis B virus (HBV) infection accounts for about 60% of the total liver cancer in developing countries and for about 23% in developed countries [3], [4]. Liver transplantation is the definitive therapy for not only resecting the tumor but also replacing the cirrhotic liver. However, only a small proportion of patients can eventually get liver transplantation, most patients exceed rigorous selection criteria or die while waiting organs [5]. Therefore, ablation or resection is the only curative treatment for most patients [6]. More than 50% of cases suffered tumor recurrence within 3 years after curative resection or ablation, not to mention the progression of chronic liver disease, which are two main causes crippling long-term survival after treatment [5], [7].

Nucleot(s)ide analogues (NA) can inhibit HBV replication, and have been shown to improve underlying liver disease and reduce the incidence of HBV-HCC [8], [9]. Therefore, NA is supposed to be able to reduce recurrence rate and improve survival after curative treatment of HBV-HCC and have been investigated in several clinical trials [10]-[22]. Some trials failed to confirm the benefit of adjuvant NA therapy [12], [16], [18]–[20], but others [10], [11], [13]–[15], [17], [21], [22], including one randomized controlled trial (RCT) [10], reported significant improvement of recurrence-free survival (RFS) or overall survival (OS).

This systematic review was implemented according to Cochrane handbook [23] and results were expressed as Hazard Ratio (HRs), which are most appropriate for survival data, taking into account not only the number but also the time of events, even further comprising the time until last follow-up for each patient who has not experienced an event [24].

## Methods

### Ethics Statement

This was a meta-analysis of published summary data and therefore did not require ethics approval.

### Criteria for considering studies for this review

Inclusion criteria: (i) Study design: both randomized controlled trials (RCT) and nonrandomized studies were considered; (ii) Study population: >18 years old, without gender restrictions, diagnosed with HBV-HCC; (iii) Therapy for HCC: curative resection or ablation; (iv) Antiviral treatment: using NA as regular therapy compared with placebo or no treatment in control group after curative therapy of HCC; (v) Initiating NA therapy: within 6 months after curative treatment; (vi) Results available on RFS or OS. Exclusion criteria: (i) Primary HCC was treated with palliative therapy (transarterial chemoembolization, radiation, systemic chemotherapy); (ii) Trials including participants co-infected with hepatitis C virus or human immunodeficiency virus.

### Search methods for identification of studies

We performed a systematic search of electronic databases (EMBASE, PubMed, Science Citation Index Expanded and Cochrane Library databases) for studies without language restriction (last literature search date: January 3, 2014). The search strategy was based on MeSH terms combining with free text words. The detailed search strategies are given in *Table S1*. Reference lists of all associated papers (relevant reviews and included studies) were checked as hand searching.

### Data collection and assessment of bias

Studies was screened according to the inclusion and exclusion criteria and data was extracted using a predesigned data extraction form by two authors independently. For duplicated publications, only the most recent or the most complete report was included. All included studies were assessed for methodological quality by two independent authors, as recommended by the Cochrane Handbook for RCTS [23] and the Newcastle-Ottawa Scale (NOS) for observational studies [25]. Any disagreement between the two authors was resolved through discussion with a third author. RFS and OS were primary outcomes. Adverse effects was secondary outcome. We would contact and request the researchers to provide key missed information.

### Statistical analysis

We performed this systematic review according to the recommendations of Cochrane Handbook [23] and reported in line with the Preferred reporting items for systematic reviews and meta-analyses (PRISMA) statement [26]. Hazard ratio (HR) between two arms was applied as a summary statistic for time-to-event outcomes like RFS and OS. HR and its standard error of each trial was calculated by a method described by Tierney and colleagues [24].

HR of individual trials were pooled into an overall HR by random-effects model. In accordance with customary, an overall HR<1 favored the NA group and the difference was considered statistically significant if the 95% CI of the HR didn't overlap 1.

Funnel plots and Begg's test would be used to evaluate the publication bias if there was sufficient studies. Sensitivity analyses were used to evaluate the reliability of the results.

Two authors input the data into RevMan 5.2 (Cochrane Collaboration, Oxford, UK) and Stata for Windows version 11.0 (StataCorp, College Station, Texas, USA), and performed all the analysis independently.

## Results

### Description of studies

**Figure 1:**
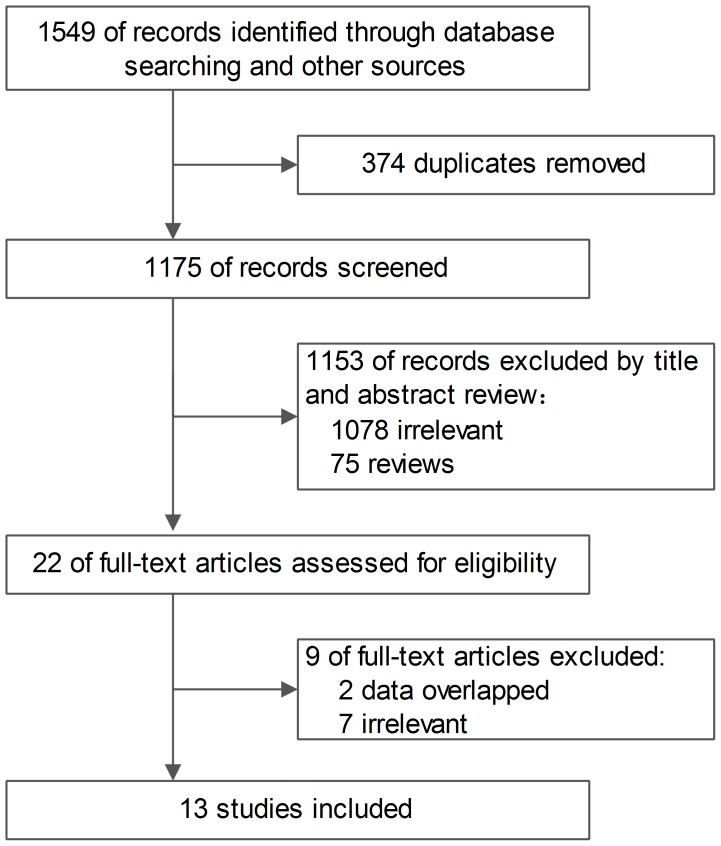
Study flow diagram.

The study screening process is shown in Figure 1. Twelve nonrandomized trials (including one large cohort) and one two-stage longitudinal study (including one RCT) were included for this systematic review (Table 1) [10]–[22]. The detailed information of the included trials was shown in *Table S2*. The reasons for excluding studies [27]–[35] were listed in *Table S3*.

**Table 1:** Characteristics of the included studies.

	Nature of Study	Sample size (T/C)	Male/Female	Age (T/C)	HCC size (cm) (T/C)	% with cirrhosis (T/C)	Curative treatment	Adjuvant treatment details	follow up (years) (T/C)	NOS (stars)	HBV-DNA (T)
**Kubo 2007**	prospective cohort	14/10, 66.7% HBeAg+	17/7	55/55	2.4/2.8	43/40	resection	LAM: 100 mg/day (with ADV rescue)	3.06/0.61	7	≥ 5000 copies/mL
**Kuzuya 2007**	retrospective cohort	16/33, 12.2% HBeAg+	41/8	59.8/61.1	NA	NA	resection or RFA	LAM: 100 mg/day (with ADV rescue)	3.2/2.7	7	≥ 400 copies/mL
**Yoshida 2008**	retrospective cohort	33/71, 18.3% HBeAg+	78/26	57/59	2.6/2.8	NA	RFA	LAM: 100 mg/day (with ADV rescue)	2.75/3.92	8	≥ 5000 copies/mL
**Chuma 2009**	retrospective cohort	20/30, 46% HBeAg+	36/14	55.6/55.7	2.1/1.7	70/83.3	resection or RFA	LAM, 100 mg/day (with ADV rescue); OR ETV, 0.5 mg/day;	2.96/4.1	8	>10^4^ copies/mL
**Koda 2009**	cohort study	22/14	NA	NA	NA	NA	resection or RFA	LAM, 100 mg/day (with ADV rescue); OR ETV, 0.5 mg/day;	NA	7	>5000 copies/mL
**Chan 2011**	retrospective cohort	42/94	105/31	57/55	9.3/9.0	74/56	resection	LAM, 100 mg/day; OR ETV, 0.5 mg/day;	NA	7	>10^5^ copies/mL
**Hann 2011**	cohort study	8/5, 30.8% HBeAg+	12/1	57/55	2.5/3.0	NA	Resection or ablation	LAM, tenofovir, ADV	5.8/1.4	9	NA
**Wu 2012**	cohort study	518/4051	3770/799	54.4/54.6	NA	48.6/38.7	resection	LAM, ETV, telbivudine	2.64/2.18	8	NA
**Lee 2012**	retrospective cohort	12/16	NA	NA	NA	100/100	resection	antiviral treatment	4.2	8	>10^4^ copies/mL
**Ke 2013**	retrospective cohort	141/141, 11% HBeAg+	256/26	48.9/49.7	4.5/5.0	81.6/81.6	resection	LAM, 100 mg/day;	2/1.9	6	≥ 200 IU/mL
**Nishikawa 2013**	retrospective cohort	65/32, 26.8% HBeAg+	67/30	56.1/60.7	2.8/3.2	58.5/46.9	resection or RFA or PCEI	LAM or ADV OR ETV	4.9/4.0	7	NA
**Su 2013**	retrospective cohort	40/142, 11% HBeAg+	158/24	52/58	NA	37.7/45.8	resection	LAM OR ETV	3.8	6	>2000 IU/mL
**Yin 2013**	cohort study	215/402, 29% HBeAg+	530/87	50/50	NA	47.0/35.8	resection	LAM: 100 mg/day (with ADV OR ETV rescue)	1.99	8	>500 copies/mL
**Yin 2013**	RCT	81/82, 41% HBeAg+	144/19	48/49	NA	24.7/28.0	resection	LAM: 100 mg/day (with ADV OR ETV rescue)	3.33	Unclear bias	> 500 copies/mL

Abbreviations: T, treated; C, control; HCC, hepatocellular carcinoma; NOS, Newcastle-Ottawa Scale; NA, not available; HBeAg+, hepatitis B virus e antigen positive; RFA, radiofrequency ablation; LAM, lamivudine; ADV, adefovir; ETV, entecavir; PCEI, percutaneous ethanol injection.

All the included studies used lamivudine as adjuvant antiviral therapy, with adefovir or entecavir rescue. A total of 6350 patients were included in this systematic review, among which 1227 were in NA-group whereas 5123 in control-group. All the studies applied resection or radiofrequency ablation (RFA) as curative treatment for primary HCC except Hann 2011 [15] and Nishikawa 2013 [22], which also used percutaneous ethanol injection (PCEI) and cryoablation besides RFA. The serum HBV-DNA levels ≥ 400 copies/mL–10, 000 copies/mL was an indication of adjuvant NA therapy in ten studies [10]-[12], [14], [16]–[22], in seven [10]–[12], [17], [19], [20], [22] of which the DNA levels in the treatment group were higher than the control group in different degrees. The risk of bias of included RCT was unclear and the NOS score was from 6 stars to 9 stars for nonrandomized trials (*Table S2*).

### Effects of intervention

**Figure 2:**
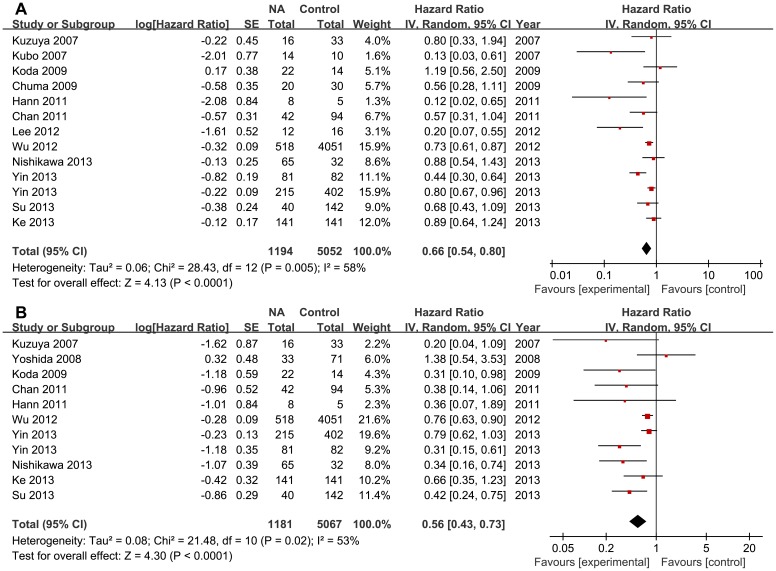
Forest plot of 13 studies on the use of NA after curative treatment of HBV-HCC. (A) Forest plot showing significant benefit of NA therapy for recurrence free survival. (B) Forest plot showing significant benefit of NA therapy for overall survival.

**Figure 3:**
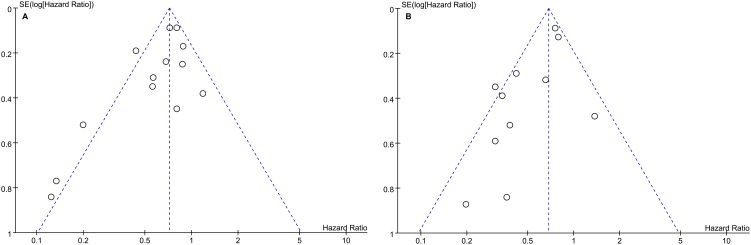
Funnel plot for assessing publication bias. (A) Funnel plot showing asymmetry indicative some extent of publication bias for recurrence free survival. (B) Funnel plot showing asymmetry indicative some extent of publication bias for overall survival.

Pooling the data of twelve studies [10]–[18], [20]–[22] that assessed RFS (Fig. 2A) in 6246 patients showed a significant difference favoring NA therapy (HR 0.66, 95% CI 0.54–0.80; p<0.0001), with significant between-study heterogeneity [χ^2^ = 28.43, degrees of freedom (df) 12; p = 0.005; I^2^ = 58%]. No significant publication bias was found by funnel plots (Fig. 3A) and Begg's test (p = 0.142). Besides, both the large cohort [13] and the RCT [10] showed significant benefit of RFS (HR 0.73, 95% CI 0.61–0.87; HR 0.44, 95% CI 0.30–0.64, respectively).

Ten studies [10]–[13], [15]–[17], [19], [20], [22] assessed OS (Fig. 2B) in 6248 patients, and showed a significant difference favoring NA therapy (HR 0.56, 95% CI 0.43–0.73; p<0.0001), with significant between-study heterogeneity (χ^2^ = 21.48, df 10; p = 0.02; I^2^ = 53%). No significant publication bias was found by funnel plots (Fig. 3B) and Begg's test (p = 0.350). Similarly, both the large cohort [13] and the RCT [10] showed significant efficacy of OS (HR 0.76, 95% CI 0.63–0.90; HR 0.31, 95% CI 0.15–0.61, respectively).

### Sensitivity analysis

Sensitivity analyses of studies with no less than 8 stars according to Newcastle Ottawa Scale, or studies with sample size greater than 50, or studies published after 2010, or after we deleted studies with lowest and highest HR or deleted studies with highest and lowest sample size, still showed significant difference between the NA group and the control group (table 2).

**Table 2:** Sensitivity analyses comparing nucleoside analogues versus control.

	No. of studies	No. of patients			HR (95% CI)	p-value	Study heterogeneity			
		NA	Control	Total			χ^2^	df	I^2^	p-value
**Studies with no less than 8 stars according to Newcastle Ottawa Scale** *										
RFS	6	854	4586	5440	0.56 (0.42, 0.76)	<0.001	18.84	5	73%	0.002
OS	5	855	4611	5466	0.70 (0.52, 0.94)	0.02	9.04	4	56%	0.06
**Excluding studies with highest and lowest HR**										
RFS	11	1164	5033	6197	0.66 (0.55, 0.80)	<0.001	22.35	10	55%	0.01
OS	9	1132	4963	6095	0.55 (0.42, 0.71)	<0.001	17.31	8	54%	0.03
**Excluding studies with highest and lowest sample size**										
RFS	11	668	996	1664	0.66 (0.52, 0.83)	<0.001	24.07	10	58%	0.007
OS	9	655	1011	1666	0.50 (0.35, 0.72)	<0.001	18.49	8	57%	0.02
**Studies with sample size greater than 50**										
RFS	8	1122	4974	6096	0.71 (0.61, 0.83)	<0.001	11.37	7	38%	0.12
OS	8	1135	5015	6150	0.60 (0.46, 0.78)	<0.001	16.94	7	59%	0.02
**Studies published after 2010**										
RFS	9	1122	4965	6087	0.66 (0.53, 0.81)	<0.001	21.36	8	63%	0.006
OS	8	1110	4949	6059	0.57(0.43, 0.74)	<0.001	15.48	7	55%	0.03

Abbreviation: No., number; HR, hazard ratio; CI, confidence interval; df, degrees of freedom; RFS, recurrence free survival; OS, overall survival.

*Yin 2013 is a two-stage longitudinal clinical study and considered as 2 studies and the randomized controlled trial (RCT) is treated as high quality of non- RCT here.

### Adverse effects

Meta-analysis comparing adverse effects of NA therapy could not be achived due to lack of enough data. Available data showed that no serious adverse effects attributable to NA therapy were recorded in nonrandomized cohort [10], [19], [20]. And in the RCT, no adverse effects caused by NA treatment were reported, except one patient who received adefovir dipivoxil plus lamivudine treatment had transient anorexia; None of the participants discontinued participation because of the adverse effects [10]. Lamivudine resistance or the emergence of YMDD mutants was from 28.6% to 37.5% but could be rescued by other types of NA or combination therapy [15], [20], [21].

## Discussion

In this systematic review, twelve nonrandomized trials (including one large cohort) and one two-stage longitudinal study (including one RCT) fulfilled our criteria. The results showed the benefit of adjuvant NA therapy for both RFS and OS, which was similar to the results of large cohort and RCT. Sensitivity analysis also confirmed the robustness of the results. The DNA levels in the treatment group were higher than the control group in seven studies [10]–[12], [17], [19], [20], [22] and the levels of alanine aminotransferase (ALT) or aspartate aminotransferase (AST) were higher than the control group in six studies [16]–[20], [22] in different degrees. Many studies have confirmed that high HBV-DNA level was a risk factor of recurrence and poorer survival after primary treatment of HCC [36]–[38], and high levels of ALT or AST indicated persistent damage to liver parenchyma, the beneficial effect of NA therapy might be blunted by the development of lamivudine resistance and the relatively higher levels of HBV-DNA, ALT or AST. Statistical assessment of side effects of NA therapy failed because only part of included studies gave general description of the common side effects other than specifying the severity and incidence. No serious adverse effects attributable to NA therapy were recorded. Lamivudine resistance or the emergence of YMDD mutants was from 28.6% to 37.5% but could be rescued by other types of NA or combination therapy. Our results were also similar to Wong 2011 [39]. But four [30], [33], [34], [40] of their nine included studies had been excluded due to our rigorous inclusion and exclusion criteria. Besides, eight more studies [10]–[16], [22] published after 2010 were included. We also gave more detailed information of adverse effects of NA therapy. This systematic review would provide more evidence for researchers and clinicians.

Since many studies have demonstrated that antiviral therapy could reduce the incidence of hepatic decompensation and the risk of HCC [41]–[43], antiviral regimens are believed to be able to decrease recurrence rate and prolong survival after curative treatment of HBV-HCC. So far, all published RCTs applied interferon (IFN) as adjuvant antiviral regimen [44]–[52] except one [10] but obtained paradoxical results. Pooled-data meta-analyses found that only hepatitis C virus-related HCC patients could benefit from adjuvant IFN therapy but HBV-HCC patients could not. Meanwhile, dose reduction and discontinuation of IFN therapy happened in a large number of patients due to adverse effects [53]. However, in this study, NA, as the first-line treatment of patients with chronic HBV infection, reduced the risk of recurrence by 36% and the risk of death by 42% in patients after curative treatment of HBV-HCC despite lamivudine resistance happened in a large proportion of patients. Newer NA, such as entecavir, tenofovir, with higher potency and minimal risk of resistance development, are most likely to make patients benefit more from adjuvant NA treatment [8], [9].

Theoretically, there is no one residual tumor cell after curative treatment of HCC. Such patients can be approximately treated as ordinary patients with chronic hepatitis B infection, except they have the highest risk of tumorigenesis. For these patients, clinicians should at least follow the indications for NA therapy in clinical practice guidelines [54]. Any trials published in the future concerning the prevention of HCC recurrence and the improvement of OS with the two current first-line agents, entecavir and tenofovir, is unlikely to be controlled, due to the great proven efficacy of them in improving viral, biochemical and pathological parameters other than HCC. It would not be ethically approved to perform placebo-controlled studies, or even lamivudine and adefovir controlled studies [8]. HBV reactivation after hepatectomy influences postoperative survival in HBV-HCC patients with preoperative low HBV-DNA levels [55]. And antiviral therapy decreases HBV reactivation in patients with HBV-HCC undergoing hepatectomy in a randomized controlled trial [56]. Further studies should be focused on broadening the indications for NA therapy after curative treatment of HBV-HCC.

Some limitations of this study should be discussed. First of all, all included studies were nonrandomized trials except one [10] and the NOS score of almost half trials was less than 8 stars. But the results showed obvious benefits of adjuvant NA therapy and were stable according to sensitivity analysis. Second, significant between-study heterogeneity existed because of the different patients (etiology, virus activity, characteristics of tumors, et al), types of NA, treatment duration, as well as interval between HCC treatment and initiation of NA therapy. In this study, we conducted the pooled data neglecting the differences but applied random-effects model. Third, some extent of publication bias was existed despite without statistical significance by Begg's test, which might indicate some kinds of report bias unpredictable. Fourth, though the serum HBV-DNA levels ≥ 400 copies/mL–10, 000 copies/mL was an indication of adjuvant NA therapy in ten studies [10]–[12], [14], [16]–[21], most of the studies did not apply exactly the same indications, making it is impossible to figure out which kind of patients can benefit more from this adjuvant therapy.

In summary, despite these limitations listed above, our study still demonstrated obvious efficacy of adjuvant NA therapy after curative treatment of HBV-HCC. Since the great proven efficacy of NA in improving viral, biochemical and pathological parameters other than HCC. It would not be ethically approved to perform any other randomized trials. Further studies should be focused on broadening the indications for NA therapy after curative treatment of HBV-HCC besides indications in clinical practice guidelines for management of chronic HBV infection.

## Supporting Information

Checklist S1 **PRISMA checklist.**
(DOC)

Table S1 **Search strategy.**
(DOC)

Table S2 **Characteristics of included studies.**
(DOC)

Table S3 **Characteristics of excluded studies.**
(DOC)
