# Supplementary material for: Nucleot(s)ide Analogues for Hepatitis B Virus-Related Hepatocellular Carcinoma after Curative Treatment: A Systematic Review and Meta-Analysis
Source: PLoS One. 2014 Jul 24;9(7):e102761. doi: 10.1371/journal.pone.0102761 (PMC4109946; doi:10.1371/journal.pone.0102761)
Supplement: Table S1 — Search strategy. (DOC) [file pone.0102761.s002.doc]

**Supplementary Table S1.** Search strategy

| **MEDLINE (PubMed),** from dates of inceptions up to January 3, 2014 |
| --- |
| #1 "Carcinoma, Hepatocellular"[Mesh]  #2 ((liver* OR hepatic OR hepatocellular* OR hepato-cellular) AND (carcinom* OR cancer* OR malign* OR tumo*)) OR HCC OR hepatoma*  #3 #1 OR #2  #4 "Antiviral Agents"[Mesh] OR "Deoxyribonucleosides"[Mesh] OR "Lamivudine"[Mesh] OR "entecavir" [Supplementary Concept] OR "adefovir" [Supplementary Concept] OR "telbivudine" [Supplementary Concept] OR "tenofovir" [Supplementary Concept]  #5 lamivudin* OR adefovir OR antivir* OR entecavir OR telbivudine OR virucid* OR virustatic* OR tenofovir OR ((nucleoside OR nucleotide) AND analogue*) OR aminoadamantane OR zidovudine  #6 #4 OR #5  #7 "Hepatitis B"[Mesh]  #8 hepatitis B OR HBV  #9 #7 OR #8  #10 "Hepatectomy"[Mesh] OR "Ablation Techniques"[Mesh]  #11 hepatectom* OR alcohol OR ethanol OR ablation OR ((liver* OR hepatocellular* OR hepatic OR hepato-cellular) AND resection)  #12 #10 OR #11  #13 survival OR death OR mortalit* OR fatality OR recurrence* OR relapse* OR recrudescence*  #14 #3 AND #6 AND #9 AND #12 AND #13 |
| **EMBASE,** from dates of inceptions up to January 3, 2014 |
| #1 'liver cell carcinoma'/exp  #2 (liver* OR hepatic OR hepatocellular* OR 'hepato cellular') AND (carcinom* OR cancer* OR malign* OR tumo*) OR hcc OR hepatoma*  #3 #1 or #2  #4 'lamivudine'/exp OR 'adefovir dipivoxil'/exp OR 'antivirus agent'/exp OR 'telbivudine'/exp OR 'tenofovir'/exp OR 'entecavir'/exp  #5 lamivudin* OR adefovir OR antivir* OR entecavir OR telbivudine OR virucid* OR virustatic* OR tenofovir OR (nucleo?ide AND analogue*) OR aminoadamantane OR ziduvudine  #6 #4 OR #5  #7 'hepatitis b'/exp  #8 (hepatitis b) OR hbv  #9 #7 OR #8  #10 'liver resection'/exp OR 'ablation therapy'/exp  #11 hepatectom* OR alcohol OR ethanol OR ablation OR ((liver* OR hepatocellular* OR hepatic OR 'hepato cellular') AND resection)  #12 #10 OR #11  #13 survival OR death OR mortalit* OR fatality OR recurrence* OR relapse* OR recrudescence*  #14 #3 AND #6 AND #9 AND #12 AND #13 |
| **Cochrane Library databases,** from dates of inceptions up to January 3, 2014 |
| #1 MeSH descriptor: [Carcinoma, Hepatocellular] explode all trees  #2 (((liver* or hepatic or hepatocellular* or hepato-cellular) and (carcinom* or cancer* or malign* or tumo*)) or HCC or hepatoma*)  #3 #1 or #2  #4 MeSH descriptor: [Lamivudine] explode all trees  #5 MeSH descriptor: [Antiviral Agents] explode all trees  #6 MeSH descriptor: [Deoxyribonucleosides] explode all trees  #7 lamivudin* or adefovir or antivir* or entecavir or telbivudine or virucid* or virustatic* or tenofovir or (nucleoside and analogue*) or aminoadamantane or ziduvudine  #8 #4 or #5 or #6 or #7  #9 MeSH descriptor: [Hepatitis B] explode all trees  #10 hbv or (hepatitis next b)  #11 #9 or #10  #12 MeSH descriptor: [Hepatectomy] explode all trees  #13 MeSH descriptor: [Ablation Techniques] explode all trees  #14 hepatectom* or alcohol or ethanol or ablation or ((liver* or hepatocellular* or hepatic or hepato-cellular) and resection)  #15 #12 or #13 or #14  #16 #3 and #8 and #11 and #15  in Cochrane Reviews (Reviews only), Other Reviews and Trials (Word variations have been searched) |
| **Science Citation Index Expanded,** from dates of inceptions up to January 3, 2014 |
| #1 TS=(((liver* OR hepatic OR hepatocellular* OR hepato-cellular) AND (carcinom* OR cancer* OR neoplasm* OR malign* OR tumo*)) OR HCC OR hepatoma*)  #2 TS=(lamivudin* OR adefovir OR antivir* OR entecavir OR telbivudine OR virucid* OR virustatic* OR tenofovir OR (nucleo?ide AND analogue*) OR aminoadamantane OR ziduvudine)  #3 TS=(hepatitis B OR hbv)  #4 TS=(hepatectom* OR alcohol OR ethanol OR ablation OR ((liver* OR hepatocellular* OR hepatic OR hepato-cellular) AND resection))  #5 TS=(survival OR death OR mortalit* OR fatality OR recurrence* OR relapse* OR recrudescence*)  #6 #5 AND #4 AND #3 AND #2 AND #1 |
