# Supplementary material for: Nucleot(s)ide Analogues for Hepatitis B Virus-Related Hepatocellular Carcinoma after Curative Treatment: A Systematic Review and Meta-Analysis
Source: PLoS One. 2014 Jul 24;9(7):e102761. doi: 10.1371/journal.pone.0102761 (PMC4109946; doi:10.1371/journal.pone.0102761)
Supplement: Table S2 — Characteristics of included studies. (DOC) [file pone.0102761.s003.doc]

**Supplementary Table S2.** Characteristics of included studies *[ordered by date of publication]*

Ke 2013

| Methods | A retrospective cohort study;  Follow-up: mean follow-up of 24 months for control group and 23 months for treated group. | |
| --- | --- | --- |
| Participation | China, single center;  282 patients (Male: 256; Female: 26; 141 in treated group, 141 in control group; Mean age: 48.9 years in treated group; 49.7 in control group);  Inclusion criteria: (1) the initial radical hepatectomy of HCC was performed in the Tumor Hospital of Guangxi Medical University. Diagnosis was verified by postoperative pathology; (2) serum Hepatitis B surface antigen (HBsAg) was positive for all patients; (3) Child-Pugh score was from 5 to 6; (4) Eastern Cooperative Oncology Group score was 0; (5) informed consent was signed.  Exclusion criteria: (1) received transarterial chemoembolization (TACE) or other antitumor therapies before operation; (2) received antiviral therapy (including interferon treatment) in the past year; (3) received prophylactic TACE or other antitumor therapies after operation; (4) combined infection of human immunodeficiency virus, hepatitis C virus, or hepatitis D virus; (5) suffered from other malignant tumors or other severe diseases simultaneously; (6) alcoholism; (7) drug abuse; and (8) pregnant or lactating women.  with cirrhosis: 81.6% in treated group, 81.6% in control group; | |
| Interventions | NA: Lamivudine [Heptodin, from GlaxoSmithKline (China) Investment Co. Ltd.] administration: orally taken by the patients once they had left the hospital or from the first week after the operation for the following one year, with a dosage of 100 mg/d;  Indications for antiviral therapy followed the Guideline of prevention and treatment for chronic hepatitis B (2010 Version)16: (1) for Hepatitis B e Antigen (HBeAg)-positive patients, the value of HBV DNA ≥105 copies/mL (equivalent to 20,000 IU/mL); or for HBeAg-negative patients, HBV DNA ≥104 copies/mL (equivalent to 2,000 IU/mL); and alanine aminotransferase (ALT) ≥ two folds the upper limit of normal; (2) for patients with compensated cirrhosis, HBV DNA ≥104 copies/mL for HBeAg-positive patients, and HBV DNA ≥103 copies/mL for HBeAg-negative patients, which does not matter whether the level of ALT is high or low; (3) for patients with cirrhosis in the decompensation period, they should receive antiviral therapy once HBV DNA is detected.  Control: no treatment;  Duration of treatment: 1 year; | |
| Outcomes | Recurrence free survival (RFS); overall survival (OS); | |
| Notes | Information about adverse events of nucleoside analogues was not available and the resistance rate of the virus and gene mutation rate were not collected. | |
| ***Assessment of Study Quality (COHORT)(6 stars)*** | | |
| **Items** | **Authors’ judgment** | **Support for judgment** |
| 1. Representativeness of the exposed cohort? | yes | A total of 478 patients meeting the aforementioned requirements were collected from January 2007 to December 2011. |
| 2. Did the non-exposed cohort draw from the same community? | yes | Among these patients, 141 treated by lamivudine after operation were integrated into the treatment group, and the remaining 337 patients without lamivudine treatment were integrated into the control group. To avoid the interference of ensured confounding factors, PSM was adopted in making group matches. Exactly 141 pairs were matched successfully. |
| 3. Ascertainment of exposure? | No | Not described. |
| 4. Outcome of interest was not present at start of study? | yes | Patients of both groups underwent radical hepatectomy for CC. orally taken by the patients once they had left the hospital or from the first week after the operation for the following one year |
| 5A. Study controls for age? | yes | To avoid the interference of ensured confounding factors, PSM was adopted in making group matches. Exactly 141 pairs were matched successfully. |
| 5B. Study controls for any additional factor? | yes | To avoid the interference of ensured confounding factors, PSM was adopted in making group matches. Exactly 141 pairs were matched successfully. |
| 6. Assessment of outcome by record linkage? | yes | Patients were followed up every two or three months after the operation for their serum HBV immune markers, HBV DNA, liver function, prothrombin time (PT), alpha-fetoprotein (AFP), ultrasonography, computed tomography or magnetic resonance imaging, and so on. Postoperative recurrence (including intrahepatic and extrahepatic recurrence) was confirmed through the appearance of intrahepatic or extrahepatic lesions meeting the features of HCC in any imaging examination. |
| 7. Follow-up long enough? | no | Mean follow-up of 24 months for control group and 23 months for treated group. |
| 8. Adequacy of Follow Up of Cohorts? | no | Not described. |

**Su 2013**

| Methods | Retrospective cohort study;  With a median follow-up of 45.9 months. | |
| --- | --- | --- |
| Participation | Taiwan, China, single center;  182 patients (40 in treated group, 142 in control group; Mean age: 52 years in treated group; 58 in control group);  The inclusion criteria were (a) positive hepatitis B surface antigen (HBsAg) in sera; (b) liver function of A or B by Child’s classification, with an indocyanine green 15-minute retention rate (ICG-15R) ,30%; (c) tumors involving no more than three Healey’s segment without portal vein main trunk involvement; (d) absence of other major diseases that might complicate surgery; (e) absence of extra-hepatic tumor dissemination.  No other forms of adjuvant anti-tumor therapy such as local ablation therapy, chemoembolization, or molecular target therapy, were performed before or after resection until the emergence of tumor recurrence. Patients with concurrent infection of hepatitis C virus (HCV) or hepatitis D virus (HDV)  were excluded.  with cirrhosis: 37.7% in treated group, 45.8% in control group; | |
| Interventions | Lamivudine/ entecavir  The criteria for the indication of reimbursed antiviral therapy for chronic hepatitis B in Taiwan were as the followings: (1) for cirrhotic patients, serum HBV DNA levels > 2000 IU/mL irrespective of serum alanine aminotransferase (ALT) levels; (2) for non-cirrhotic patients, serum ALT levels > 80 U/L in addition to serum HBV DNA levels > 20000 IU/mL in HBeAg-positive patients and HBVDNA levels > 2000 IU/mL in HBeAg-negative patients, respectively. | |
| Outcomes | Recurrence free survival (RFS); overall survival (OS); | |
| Notes | Only data of BCLC stage-A patients were extracted. | |
| ***Assessment of Study Quality (COHORT)(6 stars)*** | | |
| **Items** | **Authors’ judgment** | **Support for judgment** |
| 1. Representativeness of the exposed cohort? | yes | There were 607 consecutive treatment-naı¨ve HBV-related CC patients who underwent curative resection surgery in Taipei Veterans General Hospital from 1990 to 2007. Among them, 333 patients who had stored serum samples available for virological analysis were enrolled in this study. |
| 2. Did the non-exposed cohort draw from the same community? | Yes | As reimbursed anti-viral therapy was implemented in Taiwan since 2003; therefore, only 62 (18.6%) patients received anti-viral therapy after resection. |
| 3. Ascertainment of exposure? | no | Not described. |
| 4. Outcome of interest was not present at start of study? | yes | Yes. |
| 5A. Study controls for age? | no | The mean age in treatment group is 52 and in control group is 58 (P=0.014). |
| 5B. Study controls for any additional factor? | no | There are still several other imbalanced factors. |
| 6. Assessment of outcome by record linkage? | yes | After surgery, patients visited outpatient clinics regularly every three months and assessed by testing serum liver biochemistries and AFP levels, and ultrasonography. |
| 7. Follow-up long enough? | yes | With a median follow-up of 45.9 months. |
| 8. Adequacy of Follow Up of Cohorts? | yes | There is not any evidence to show the follow up is not adequate. |

**Nishikawa 2013**

| Methods | Retrospective cohort study;  The median observation periods were 4.9 years (range, 0.2–11.9) for NA group and 4.0 years (range, 1.1–10.4) for control group. | |
| --- | --- | --- |
| Participation | Treatment-naïve HBV-related HCC patients received curative therapy at our institution between January 2001 and November 2012. All patients were positive for hepatitis B surface antigen (HBsAg) and negative for anti-HCV (HCVAb). Curative therapy was defined as therapy resulting in no apparent viable tumor on dynamic computed tomography (CT) performed within 1 month after initial treatment for HCC.  Patients in control group did not receive NA therapy for the following reasons: (i) sustained low HBV viral load during the follow-up period (n = 20); (ii) informed consent for NA therapy was not obtained due to economic reasons (n = 7); and (iii) unknown reasons (n = 5).  Inclusion criteria: HBV-related hepatocellular carcinoma (HCC) who were curatively treated with resection or radiofrequency ablation (RFA); (1) solitary tumor less than 5 cm in diameter or 2-3 tumors with the largest one no more than 3 cm in diameter, (2) positive for serum hepatitis B surface antigen (HBsAg) and negative for hepatitis C virus antibody (HCVAb).  Exclusion criteria: anti-HCV seropositive, co-infection of HBV and HCV, non HBV and HCV;;  characteristics of nodules: single tumor, 65%; tumor size: 2.8 cm in NA group, 3.2 cm in control group; | |
| Interventions | Nucleotide Analogues (NA) | |
| Outcomes | Recurrence free survival (RFS); overall survival (OS); | |
| Notes | In the current study, the baseline characteristics in the two groups were not well balanced for survival analysis, leading to bias. | |
| | ***Assessment of Study Quality (COHORT)(7 stars)*** | | --- | | | |
| **Items** | **Authors’ judgment** | **Support for judgment** |
| 1. Representativeness of the exposed cohort? | yes | Obviously representative series of cases. |
| 2. Did the non-exposed cohort draw from the same community? | yes | Hospital controls, within same community. |
| 3. Ascertainment of exposure? | yes | By medical records. |
| 4. Outcome of interest was not present at start of study? | yes | HBV-related hepatocellular carcinoma (HCC) who were curatively treated with resection or ablation. |
| 5A. Study controls for age? | no | Obviously not. |
| 5B. Study controls for any additional factor? | no | Obviously not. |
| 6. Assessment of outcome by record linkage? | yes | Medical records. |
| 7. Follow-up long enough? | yes | Probably yes. |
| 8. Adequacy of Follow Up of Cohorts? | yes | Probably yes. |

**Yin 2013**

| Methods | two-stage longitudinal study that included a randomized clinical trial (RCT) and a cohort study;  The median follow-up duration was 23.83 months for all patients (interquartile range, 13.29 to 32.15 months) in cohort study and 39.93 months (interquartile range, 27.27 to 47.80 months) in RCT.  This was an open-label RCT undertaken between July 2007 and July 2009. Serologic examinations before surgery and diagnostic criteria were the same as those for the nonrandomized cohort. The study statistician generated 200 randomization codes. The ratio of test to control was 1:1 and the block size was 4. Patients were randomly assigned to either antiviral or control arms according to enrollment sequence. Sample size was estimated on the basis of 2-year recurrence rates of 77% in the control group and 52% in the antiviral group in our nonrandomized cohort, with patients recruited from May 2006 to June 2007 as the expected difference. The minimum sample size was 75 for each group (two-sided α = 0.05; β = 0.10; power, 90%). | |
| --- | --- | --- |
| Participation | 617 in cohort study (215 in treatment group; 402 in control group); 163 in RCT (81 in treatment group; 82 in control group);  The patients enrolled onto the RCT were not included in the nonrandomized cohort.  The nonrandomized cohort initially enrolled 896 patients with HCC who were diagnosed as previously described and who received radical hepatectomy from May 2006 to June 2009. Of those, 795 were seropositive for HBsAg. We excluded the patients coinfected with HCV, those who recurred within 1 month after surgery, and those lost to follow-up (contact information was lost for 91.5% of these patients). Finally, 617 previously untreated patients with HCC with serum HBV DNA of more than 500 copies/mL were enrolled onto this stage. Of those, 215 received postoperative NA treatments.  For RCT:  Inclusion criteria: 1. Age 18~70years, without gender stricture; 2. HBsAg (+) and HBV-DNA>500 copies/mL; 3. Liver function: Child A or B; 4. Without surgical contradiction and life span is longer than half a year; 5. Without HCV, HIV or syphilis infection; 6. After redial excision; 7. Participants attend the trial of their own free will and sign the informed consent form before recruiting.  Exclusion criteria：1. Pregnant or breast-feeding women or possible to conceive again; 2. With mental disorder or central nervous abnormality or episode; 3. With abnormal ECG or heart disease including congestive heart failure, coronary heart disease, arrhythmia and myocardial infarction; 4. With serious infection, sepsis, diabetes and metabolic disorder; 5. With active digestive ulcer and defect of defect of absorption; 6. History of malignant or metastatic tumor in other sites in last 5 years; 7 Patients who is participating in other trials; 8. Other conditions are not proper for the trial. | |
| Interventions | The patients began receiving oral NAs within 1 week after surgery until HBsAg seroconversion. Lamivudine (100 mg per day; GlaxoSmithKline, Beijing, China) was used as the choice antiviral treatment. Adefovir dipivoxil (10 mg per day; GlaxoSmithKline) plus lamivudine or entecavir (0.5 mg per day; Sino-American Squibb, Shanghai, China) was used if the patients were drug resistant. The dosage was adjusted in some patients according to their creatinine clearance rates. | |
| Outcomes | Recurrence free survival (RFS); overall survival (OS); side effect. | |
| Notes | Patients with microscopic vascular invasion are beyond 30% in cohort study and 10% in RCT;  We stratified the study patients by each of the five imbalanced variables between the antiviral and control arms and evaluated the effect of antiviral treatment on postoperative prognosis in each stratum. It was found that antiviral treatment did not significantly increase RFS and OS in those with grade 1 to 2 tumor differentiation and OS in those with AFP ≤ 20 ng/mL.  No serious adverse effects caused by NA treatment were reported.  No adverse effects caused by NA treatment were reported, except one patient who received adefovir dipivoxil plus lamivudine treatment had transient anorexia. None of the participants discontinued participation in the RCT because of the adverse effects. | |
| ***Assessment of Study Quality (COHORT)(8 stars)*** | | |
| **Items** | **Authors’ judgment** | **Support for judgment** |
| 1. Representativeness of the exposed cohort? | yes | A total of 1,096 consecutive patients with HCC who received radical hepatectomy at the Department of Comprehensive Surgery, Eastern Hepatobiliary Surgery Hospital, from May 2006 to July 2009. |
| 2. Did the non-exposed cohort draw from the same community? | yes | Finally, 617 previously untreated patients with HCC with serum HBV DNA of more than 500 copies/mL were enrolled onto this stage. Of those, 215 received postoperative NA treatments. |
| 3. Ascertainment of exposure? | yes | All patients enrolled onto this study were re-examined at our hospital within 1 month after surgery. The follow-up examination was performed at our outpatient clinic every 3 to 6 months or sometimes at outpatient clinics at local hospitals if the patients had related symptoms. |
| 4. Outcome of interest was not present at start of study? | yes | Obviously. |
| 5A. Study controls for age? | yes | Without any obvious difference. |
| 5B. Study controls for any additional factor? | yes | We stratified the study patients by each of the five imbalanced variables between the antiviral and control arms and evaluated the effect of antiviral treatment on postoperative prognosis in each stratum. |
| 6. Assessment of outcome by record linkage? | yes | All patients enrolled onto this study were re-examined at our hospital within 1 month after surgery. The follow-up examination was performed at our outpatient clinic every 3 to 6 months or sometimes at outpatient clinics at local hospitals if the patients had related symptoms, according to standard epidemiologic procedure. Computed tomography and/or magnetic resonance imaging were conducted for our patients every 6 months or if HCC recurrence was suspected to confirm the diagnosis. |
| 7. Follow-up long enough? | no | The median follow-up duration was 23.83 months for all patients (interquartile range, 13.29 to 32.15 months) in cohort study. |
| 8. Adequacy of Follow Up of Cohorts? | yes | Patients lost to . |
| ***Risk of bias (RCT)(***Unclear***)*** | | |
| **Bias** | **Authors’ judgment** | **Support for judgment** |
| Random sequence generation (selection bias) | Low risk | The study statistician generated 200 randomization codes. The ratio of test to control was 1:1 and the block size was 4. |
| Allocation concealment (selection bias) | Unclear risk | Patients were randomly assigned to either antiviral or control arms according to enrollment sequence. |
| Blinding of participants and personnel (performance bias) | Low risk | The outcome is not likely to be influenced by lack of blinding. |
| Blinding of outcome assessment (detection bias) | Low risk | The outcome is not likely to be influenced by lack of blinding. |
| Incomplete outcome data (attrition bias) | Unclear risk | 8 patients in treatment group and 9 patients in control group were ruled out during the analysis. Though the reasons were similar between groups, the influence was unclear. |
| Selective reporting (reporting bias) | Low risk | It is clear that the published reports include all expected outcomes. |
| Other bias | Low risk | No additional biases |

**Wu 2012**

| Methods | A nationwide cohort study;  Follow-up: mean follow-up of 2.18 years for control group and 2.64 years for treated group; | |
| --- | --- | --- |
| Participation | Taiwan, nationwide;  4569 patients (Male: 3770; Female: 799; 518 in treated group, 4051 in control group; Mean age: 54.4 years in treated group; 54.6 in control group);  Inclusion criteria: newly diagnosed with HBV-related HCC and who received curative liver resection;  Exclusion criteria: if diagnosed with hepatitis C, other viral hepatitis, malignant tumor, or if they received antiviral treatments for more than 3 months before the index admission; if they received liver resection, transarterial chemoembolization, percutaneous ethanol injection, radiofrequency ablation, or liver transplantation before the index hospitalization;  with cirrhosis: 48.6% in treated group, 38.7% in control group; | |
| Interventions | NA: 487 patients received only 1 nucleoside analogue, including 159 patients who received lamivudine, 292 patients who received entecavir, and 36 patients who received telbivudine. The main patients received more than 1 nucleoside analogue;  Control: no treatment;  Duration of treatment: The mean (SD) duration of nucleoside analogue use in treated patients was 1.45 (1.38) years and the median (IQR) was 0.95 (0.48-1.94) years; | |
| Outcomes | Recurrence free survival (RFS); overall survival (OS); | |
| Notes | Information about adverse events of nucleoside analogues was not available from the NHIRD. | |
| ***Assessment of Study Quality (COHORT)(8 stars)*** | | |
| **Items** | **Authors’ judgment** | **Support for judgment** |
| 1. Representativeness of the exposed cohort? | yes | We identified all hospitalized patients who were admitted with a primary diagnosis of HCC for the first time and who received curative liver resection between October 1, 2003, and September 30, 2010. |
| 2. Did the non-exposed cohort draw from the same community? | yes | The untreated cohort comprised patients who never received NA and the treated cohort was patients who received nucleoside analogues for at least 90 days. |
| 3. Ascertainment of exposure? | yes | Information regarding patients’ medications was retrieved from the pharmacy prescription database. Reliability of the retrieved information was verified independently by 2 statisticians. |
| 4. Outcome of interest was not present at start of study? | yes | Those receiving antiviral treatments for less than 90 days during the observation period or prior to the observation period were excluded. |
| 5A. Study controls for age? | yes | To determine the independent risk factors for HCC recurrence, multivariable analyses and stratified analyses using hazard ratios (HRs) were carried out with modified Cox proportional hazards models in the presence of competing risk event after adjusting for age, sex, resection modality, liver cirrhosis, diabetes, propensity score, and use of statins, NSAIDs or aspirin, and metformin. |
| 5B. Study controls for any additional factor? | yes | To determine the independent risk factors for HCC recurrence, multivariable analyses and stratified analyses using hazard ratios (HRs) were carried out with modified Cox proportional hazards models in the presence of competing risk event after adjusting for age, sex, resection modality, liver cirrhosis, diabetes, propensity score, and use of statins, NSAIDs or aspirin, and metformin. |
| 6. Assessment of outcome by record linkage? | yes | Hepatocellular carcinoma recurrence was defined as rehospitalization with a primary diagnosis of HCC after the index admission date and a treatment modality for HCC recurrence, such as surgery, transarterial chemoembolization, percutaneous ethanol injection, radiofrequency ablation, or liver transplantation during the study period. |
| 7. Follow-up long enough? | no | mean follow-up of 2.18 years for control group and 2.64 years for treated group |
| 8. Adequacy of Follow Up of Cohorts? | yes | It is nearly impossible for these HCC patients to withdraw from the NHI program before death. Therefore, there were no missing data or loss of follow-up in our study population. |

**Lee 2012**

| Methods | Retrospective cohort study; | |
| --- | --- | --- |
| Participation | We retrospectively reviewed the medical records of 70 cirrhotic patients with curative resection of HBV related HCC from January 2000 to March 2010. Of 28 patients with HBV DNA level more than 10^4^ copies/mL, 12 patients received antiviral therapy after curative resection. There was no significant difference in clinical pathologic features between the two groups.  With the median observation period of 50.4 months. | |
| Interventions | Twelve months of antiviral therapy after curative resection of HBV-related HCC. | |
| Outcomes | Recurrence free survival (RFS) and late recurrence (>1 year); HBV DNA suppression rate. | |
| Notes | The proportion of censored data between the two groups is inconsistent. | |
| ***Assessment of Study Quality (COHORT)(8 stars)*** | | |
| **Items** | **Authors’ judgment** | **Support for judgment** |
| 1. Representativeness of the exposed cohort? | yes | We retrospectively reviewed the medical records of 70 cirrhotic patients with curative resection of HBV related HCC from January 2000 to March 2010. Of 28 patients with HBV DNA level more than 10^4^ copies/mL, 12 patients received antiviral therapy after curative resection. There was no significant difference in clinical pathologic features between the two groups. |
| 2. Did the non-exposed cohort draw from the same community? | Yes | Drawn from the same community as the exposed cohort. |
| 3. Ascertainment of exposure? | yes | Medical records. |
| 4. Outcome of interest was not present at start of study? | yes | Yes. |
| 5A. Study controls for age? | yes | There was no significant difference in clinical pathologic features between the two groups. |
| 5B. Study controls for any additional factor? | yes | There was no significant difference in clinical pathologic features between the two groups. |
| 6. Assessment of outcome by record linkage? | yes | By medical records. |
| 7. Follow-up long enough? | yes | With the median observation period of 50.4 months. |
| 8. Adequacy of Follow Up of Cohorts? | no | The proportion of censored data between the two groups is inconsistent. |

**Hann 2011**

| Methods | A cohort study; | |
| --- | --- | --- |
| Participation | Diagnosis of HCC was made by magnetic resonance imaging (MRI), alpha fetoprotein (AFP) or histology. Fifteen CHB patients with a single HCC (4 cm, the size defined as locally curable) received local ablation and were judged to have a complete response to one of the following modalities; resection, cryoablation, RFA and PCEI. All were HBs-Ag positive, anti-HCV negative and Asian Americans. None received antiviral therapy before HCC diagnosis.  Six patients, diagnosed between 1991 and 1997 who received no antiviral therapy were considered the historical controls.  All underwent local tumor ablation and were considered to have successful elimination. | |
| Interventions | Nine patients diagnosed between 2000 and 2004 received antiviral therapy immediately at diagnosis, initially with LAM later with tenofovir (TDF) which was available off-label in 2001, and with adefovir (ADV) in 2002. Decision to add ADV or TDF was based on LAM resistance defined by the virologic breakthrough. | |
| Outcomes | Recurrence free survival (RFS); overall survival (OS); LAM resistance. | |
| Notes | Three patients developed LAM resistance at months 22, 24, and 27 and placed on combination therapy.  The patients in untreated group were historical controls, which might introduce a certain degree of bias. | |
| ***Assessment of Study Quality (COHORT)(9 stars)*** | | |
| **Items** | **Authors’ judgment** | **Support for judgment** |
| 1. Representativeness of the exposed cohort? | yes | Nine patients diagnosed between 2000 and 2004 received antiviral therapy immediately at diagnosis. |
| 2. Did the non-exposed cohort draw from the same community? | yes | Six patients, diagnosed between 1991 and 1997 who received no antiviral therapy were considered the historical controls. |
| 3. Ascertainment of exposure? | yes | Nine patients diagnosed between 2000 and 2004 received antiviral therapy immediately at diagnosis, initially with LAM later with tenofovir (TDF) which was available off-label in 2001, and with adefovir (ADV) in 2002. Decision to add ADV or TDF was based on LAM resistance defined by the virologic breakthrough. |
| 4. Outcome of interest was not present at start of study? | yes | All underwent local tumor ablation and were considered to have successful elimination. |
| 5A. Study controls for age? | yes | Median age, size of HCC and AFP levels between the two are similar. |
| 5B. Study controls for any additional factor? | yes | Median age, size of HCC and AFP levels between the two are similar. |
| 6. Assessment of outcome by record linkage? | yes | MRI was obtained 1 month after ablation and at 3 month intervals subsequently. All patients on antiviral therapy have maintained undetectable HBV DNA and are being followed at 3–4 monthly intervals while on therapy. |
| 7. Follow-up long enough? | yes | By this time of report, two longest survivors are alive beyond 10 years and five are alive and well between 6 and 9 years. |
| 8. Adequacy of Follow Up of Cohorts? | yes | All patients were followed long enough. |

**Chan 2011**

| Methods | A cohort study; | |
| --- | --- | --- |
| Participation | Queen Mary Hospital, Hong Kong Special Administrative Region;  From September 1, 2003, through December 31, 2007, 379 patients underwent hepatectomy for HCC in the Department of Surgery at our institution. Among them, 136 patients had chronic HBV infection. Initiation of antiviral therapy within 12 months after hepatectomy was based on the following criteria: (1) alanine aminotransferase level more than 2 times the upper limit of reference values, with or without a serum HBV DNA level greater than 10^5^ copies/mL; (2) serum alanine aminotransferase level greater than the upper limit of the reference value but less than 2 times the value, with serum HBV DNA level greater than 10^5^ copies/mL; or (3) liver biochemistry findings within the reference range, with serum HBV DNA levels greater than 10^5^ copies/ mL only. None of the patients developed tumor recurrence before the initiation of antiviral therapy. Patients who received antiviral therapy after hepatectomy were categorized as the treatment group, and those who did not were categorized as the control group. | |
| Interventions | During the early study period, lamivudine (100 mg/d) was used as our first-choice antiviral treatment; however, in recent years, we have used entecavir (0.5 mg/d) because of its high potency against HBV DNA activity.  Antiviral therapy was started in 42 patients (lamivudine in 38 and entecavir in 4) at a median of 8 days (range, 0-12 months) after hepatectomy, whereas 94 patients did not receive any antiviral treatment. | |
| Outcomes | Disease-free and overall survival rates | |
| Notes | There were significantly more patients with Child-Pugh class B cirrhosis in the control group.  None of the patients developed tumor recurrence before the initiation of antiviral therapy, which might lead to the patients with early recurrence divided into control group and the relatively bad outcome of the control group. | |
| ***Assessment of Study Quality (COHORT)(7 stars)*** | | |
| **Items** | **Authors’ judgment** | **Support for judgment** |
| 1. Representativeness of the exposed cohort? | yes | From September 1, 2003, through December 31, 2007, 379 patients underwent hepatectomy for HCC in the Department of Surgery at our institution. Among them, 136 patients had chronic HBV infection. |
| 2. Did the non-exposed cohort draw from the same community? | No | Initiation of antiviral therapy within 12 months after hepatectomy was based on the following criteria: (1) alanine aminotransferase level more than 2 times the upper limit of reference values, with or without a serum HBV DNA level greater than 105 copies/mL; (2) serum alanine aminotransferase level greater than the upper limit of the reference value but less than 2 times the value, with serum HBV DNA level greater than 105 copies/mL; or (3) liver biochemistry findings within the reference range, with serum HBV DNA levels greater than 105 copies/ mL only. |
| 3. Ascertainment of exposure? | yes | Antiviral therapy was started in 42 patients (lamivudine in 38 and entecavir in 4) at a median of 8 days (range, 0-12 months) after hepatectomy. |
| 4. Outcome of interest was not present at start of study? | yes | None of the patients developed tumor recurrence before the initiation of antiviral therapy. |
| 5A. Study controls for age? | yes | There was no significant difference in the age, distribution of sex, and the incidence of screening-detected HCC between the 2 groups of patients. |
| 5B. Study controls for any additional factor? | No | There were significantly more patients with Child-Pugh class B cirrhosis in the control group. |
| 6. Assessment of outcome by record linkage? | yes | All patients were available for follow-up. Computed tomography of the liver was performed at 1 month after hepatectomy to confirm complete tumor clearance and then every 3 months for surveillance. Blood tests for liver biochemistry values, clotting profile, complete hematological profile, and serum AFP  levels were also determined at 1 month after hepatectomy and then at 3-month intervals. Liver biochemistry findings at the time of tumor recurrence were recorded. |
| 7. Follow-up long enough? | yes | Most patients have been followed for more than 5 years. |
| 8. Adequacy of Follow Up of Cohorts? | yes | All patients were available for follow-up. |

**Koda 2009**

| Methods | Cohort study; | |
| --- | --- | --- |
| Participation | 36 patients who underwent initial curative treatment, 22 patients in the nucleotide analog group (5 treated by hepatic resection and 17 by RFA) and 14 patients in the control group (7 by hepatic resection and 7 by RFA).  Inclusion criteria: 1) HBV-DNA in serum more than 3.7 LGE/mL, 2) serum ALT more than 40 IU/L, 3) HCC underwent curative treatment.  The mean follow-up period in all patients was 28.6 ± 16.7 months for the nucleotide analog group and 36.3 ± 21.6 for the control group. | |
| Interventions | lamivudine (100 mg/day) or entecavir (0.5 mg/day);  Control: no treatment;  Duration of treatment: during the observation period; | |
| Outcomes | changes in liver function, HCC recurrence and survival rate | |
| Notes | none | |
| ***Assessment of Study Quality (COHORT)(7 stars)*** | | |
| **Items** | **Authors’ judgment** | **Support for judgment** |
| 1. Representativeness of the exposed cohort? | yes | Consecutive or obviously representative series of cases. |
| 2. Did the non-exposed cohort draw from the same community? | yes | Hospital controls. |
| 3. Ascertainment of exposure? | yes | Medical records. |
| 4. Outcome of interest was not present at start of study? | no | Without definition of curative treatment of HCC. |
| 5A. Study controls for age? | yes | The difference between the two groups was not significant. |
| 5B. Study controls for any additional factor? | yes | The difference between the two groups was not significant. |
| 6. Assessment of outcome by record linkage? | yes | We examined the clinical features such as ascites and hepatic encephalopathy by physical findings and laboratory tests every 1-3 months. |
| 7. Follow-up long enough? | no | The mean follow-up period in all patients was 28.6 ± 16.7 months for the nucleotide analog group and 36.3 ± 21.6 for the control group. |
| 8. Adequacy of Follow Up of Cohorts? | yes | All patients were followed using abdominal sonography or computed tomography (CT) every 3 months as well as the measurement of tumor markers, serum alpha-fetoprotein (AFP) and des-gamma-carboxyl prothrombin (DCP) every 1-3 months. |

**Chuma 2009**

| Methods | Retrospective cohort.  Follow-up: 49.2 (12–89) months in control group; 35.5 (12–67) months in treatment group | |
| --- | --- | --- |
| Participation | Japan, single center;  30 patients in control group(Male: 22; Female: 8) and 20 patients in treatment group (Male: 14; Female: 6);  Inclusion criteria: (1) hepatic resection or RFA for initial HCC treatment; (2) three or fewer lesions, each 3 cm or less in diameter; (3) no extrahepatic metastasis or vascular invasion; (4) curative treatment and no local recurrence after treatment; (5) no recurrence 3 months after treatment; (6) liver function of Child-Pugh class A or B; (7) no excessive alcohol intake ([65 g/day); and (8) no evidence of any other active neoplastic site; (9) high serum HBV DNA levels ([4 log10] copies/mL). | |
| Interventions | Seventeen patients received antiviral therapy within 1 month after HCC treatment. The remaining three patients received antiviral therapy from diagnosis of active viral hepatitis B; the intervals between HCC treatment and the commencement of nucleotide analogue in these three patients were 12, 15, and 22 months. seven patients received lamivudine only (100 mg/day). Entecavir alone (0.5 mg/day) was used in five patients. Adefovir dipivoxil (10 mg/day) was used together with lamivudine to suppress lamivudine-resistant hepatitis B virus (HBV) in eight patients. | |
| Outcomes | Recurrence free survival (RFS); overall survival (OS); | |
| Notes | The remaining three patients received antiviral therapy from diagnosis of active viral hepatitis B; the intervals between HCC treatment and the commencement of nucleotide analogue in these three patients were 12, 15, and 22 months. | |
| ***Assessment of Study Quality (COHORT)(8 stars)*** | | |
| **Items** | **Authors’ judgment** | **Support for judgment** |
| 1. Representativeness of the exposed cohort? | yes | Consecutive or obviously representative series of cases. |
| 2. Did the non-exposed cohort draw from the same community? | yes | Hospital controls. |
| 3. Ascertainment of exposure? | yes | Medical records. |
| 4. Outcome of interest was not present at start of study? | yes | no recurrence 3 months after treatment; |
| 5A. Study controls for age? | yes | Univariate and multivariate analysis of the risk ratios for the recurrence of HCC were performed using Cox’s proportional hazards regression analysis. The risk factors examined included gender, age, HBeAg status, ALT, platelet count, PT, albumin, bilirubin, liver fibrosis, tumor differentiation, AFP, PIVKA-II, tumor size, tumor number, and initial treatment. |
| 5B. Study controls for any additional factor? | yes | Univariate and multivariate analysis of the risk ratios for the recurrence of HCC were performed using Cox’s proportional hazards regression analysis. The risk factors examined included gender, age, HBeAg status, ALT, platelet count, PT, albumin, bilirubin, liver fibrosis, tumor differentiation, AFP, PIVKA-II, tumor size, tumor number, and initial treatment. |
| 6. Assessment of outcome by record linkage? | yes | During follow-up, clinical evaluations and biochemical tests were performed every 1–3 months. Patients underwent triphasic computed tomography of the liver every 3 months. The endpoint used in this study was the recurrence of HCC. |
| 7. Follow-up long enough? | yes | The mean follow-up period for all patients was 40 (12–92) months. |
| 8. Adequacy of Follow Up of Cohorts? | no | Without description. |

**Yoshida 2008**

| Methods | Retrospective cohort.  Follow-up: 47 ± 22 months in control group; 33 ± 20 months in treatment group | |
| --- | --- | --- |
| Participation | Japan, single center;  HBs antigen was positive in 104 patients and all of them received curative RFA therapy. 33 patients (male/female: 23:10) received LAM after ablation therapy. the rest 71 patients (male/female: 55:16)did not.  Inclusion criteria: HBs antigen was positive; received curative RFA therapy; The effectiveness of ablation was evaluated with contrast-enhanced computed tomography in each patient. | |
| Interventions | after RFA treatment was at the discretion of each patient and the physician in charge on discussing merits and demerits of the therapy. When indicated, LAM was given at a dose of 100 mg per day orally after obtaining written informed consent. | |
| Outcomes | Overall and recurrence-free survival; liver function; Antiviral efficacy | |
| Notes | No adverse effects attributable to LAM were recorded. | |
| ***Assessment of Study Quality (COHORT)(8 stars)*** | | |
| **Items** | **Authors’ judgment** | **Support for judgment** |
| 1. Representativeness of the exposed cohort? | yes | Consecutive or obviously representative series of cases. |
| 2. Did the non-exposed cohort draw from the same community? | yes | Hospital controls. |
| 3. Ascertainment of exposure? | yes | Medical records. |
| 4. Outcome of interest was not present at start of study? | yes | The effectiveness of ablation was evaluated with contrast-enhanced computed tomography in each patient. |
| 5A. Study controls for age? | yes | The propensity score for LAM administration was calculated by using a logistic regression model with LAM administration as the dependent variable and sex, age, liver function (represented by Child-Pugh score), and HCC stage as the independent variables. A control was selected for each patient who received LAM by using the propensity score as the matching variable, the maximum distance of which was set at 0.1. |
| 5B. Study controls for any additional factor? | yes | The propensity score for LAM administration was calculated by using a logistic regression model with LAM administration as the dependent variable and sex, age, liver function (represented by Child-Pugh score), and HCC stage as the independent variables. A control was selected for each patient who received LAM by using the propensity score as the matching variable, the maximum distance of which was set at 0.1. |
| 6. Assessment of outcome by record linkage? | yes | Recurrence of HCC was monitored with ultrasonography and computed tomography every 3–4 months. |
| 7. Follow-up long enough? | yes | Follow-up: 47 ± 22 months in control group; 33 ± 20 months in treatment group. |
| 8. Adequacy of Follow Up of Cohorts? | no | Without description. |

**Kuzuya 2007**

| Methods | Retrospective cohort.  Follow-up: 32.6 ± 18.9 months in control group; 38.0 ± 21.6 months in treatment group | |
| --- | --- | --- |
| Participation | Japan, single center;  49 patients (41 men and 8 women) (mean age: 60.6 ± 9.2 years).  inclusion criteria: (i) patients who did not receive lamivudine therapy prior to diagnosis of initial HCC; (ii) patients who underwent hepatic resection or RFA for initial HCC treatment; and (iii) patients who were judged as having complete curative response 1 month after initial HCC treatment. (iv) positive for hepatitis B surface antigen (HBsAg) and were not positive for hepatitis C virus antibody. | |
| Interventions | 16 received lamivudine (Zeffix, Glaxo-Smith-Kline, UK) at a dose of 100 mg/day (lamivudine group) for as long as possible. The remaining 33 patients did not receive lamivudine (control group).  In the lamivudine group, the mean lamivudine treatment period  was 22.7 ± 14.2 months (range 6.3–54.8). | |
| Outcomes | changes in remnant liver function, HCC recurrence and survival | |
| Notes | There was a significant difference with respect to HBV-DNA among the two groups. Median HBV-DNA levels in the lamivudine group (6.2 log copies/mL, range 2.8–8.3) were significantly higher than those in the control group (4.1 log copies/mL, range 2.6–7.1) (P = 0.003).  The emergence of YMDD mutants was observed in five of 16 patients in the lamivudine group (31.6%). There were no serious adverse effects during lamivudine therapy. | |
| ***Assessment of Study Quality (COHORT)(7 stars)*** | | |
| **Items** | **Authors’ judgment** | **Support for judgment** |
| 1. Representativeness of the exposed cohort? | yes | Between December 1998 and December 2004, a total of 105 patients with chronic HBV infection were diagnosed as having initial HCC (not recurrence) and were treated at the Department of Gastroenterology, Nagoya University School of Medicine or the Department of Gastroenterology, Ogaki Municipal Hospital. Of 105 patients, 49 patients meeting the inclusion criteria were enrolled. |
| 2. Did the non-exposed cohort draw from the same community? | yes | Hospital controls. |
| 3. Ascertainment of exposure? | yes | Medical records. |
| 4. Outcome of interest was not present at start of study? | yes | Dynamic computed tomography (CT) was performed at 1 month after initial treatment of HCC in all patients in order to assess the therapeutic effects; no enhancement in the treated area was considered to indicate complete curative response. |
| 5A. Study controls for age? | yes | There were no significant differences among the two groups with regard to age, sex, HBeAg, ALT, PT, albumin, total bilirubin, platelet count, presence of ascites, hepatic encephalopathy, Child–Pugh score, stage of initial HCC, initial HCC treatment and follow-up period. |
| 5B. Study controls for any additional factor? | yes | There were no significant differences among the two groups with regard to age, sex, HBeAg, ALT, PT, albumin, total bilirubin, platelet count, presence of ascites, hepatic encephalopathy, Child–Pugh score, stage of initial HCC, initial HCC treatment and follow-up period. |
| 6. Assessment of outcome by record linkage? | yes | All patients were followed primarily with abdominal US and liver function tests, as well as measurement of tumor markers, serum a-fetoprotein and des-gamma-carboxy prothrombin, at 1- to 3-month intervals after initial treatment for HCC. When suspicious findings on US or tumor markers were detected, dynamic CT was performed in order to examine recurrent HCC. Angiography assisted CT was performed whenever possible. |
| 7. Follow-up long enough? | no | Follow-up: 32.6 ± 18.9 months in control group; 38.0 ± 21.6 months in treatment group. |
| 8. Adequacy of Follow Up of Cohorts? | no | Without description. |

Kubo 2007

| Methods | Cohort study.  Follow-up: The median follow up from operation until the detection of HCC recurrence or the study endpoint (30 April 2006) in this study was 759 days (34–2053). The median follow up for each group was 1117 days (187–2037) for patients receiving lamivudine and 224 days (34–2053) for the controls. | |
| --- | --- | --- |
| Participation | Japan, single center;  From November 2000 to October 2005, curative resection of HCC was performed at Osaka City University Hospital in 24 patients seropositive for HB surface antigen (HBsAg) who were negative for anti-hepatitis C virus antibody and had high serum concentrations of HBV DNA. The patients had not received any lamivudine therapy before the operation. the serum concentration of HBV DNA was at least 3.7 logarithms of the genome equivalent (LGE) per milliliter.  24 patients (17 men and 7 women) (mean age: 55 years). | |
| Interventions | Lamivudine therapy was started in the 14 who then agreed to this therapy and gave their informed consent (lamivudine group), while the control group consisted of the other 10 patients who declined treatment with the drug because of the possibility of adverse events or the necessity of long-term administration of the drug.  Fourteen patients received lamivudine therapy (100 mg/day), beginning 2 weeks to 2 months after surgery. The period of lamivudine administration was 6 months to 65 months (mean, 32 months). | |
| Outcomes | changes in remnant liver function, HCC recurrence | |
| Notes | In four patients YMDD mutant viruses were detected after beginning lamivudine administration. | |
| ***Assessment of Study Quality (COHORT)(7 stars)*** | | |
| **Items** | **Authors’ judgment** | **Support for judgment** |
| 1. Representativeness of the exposed cohort? | yes | From November 2000 to October 2005, curative resection of HCC was performed at Osaka City University Hospital in 24 patients seropositive for HB surface antigen (HBsAg) who were negative for anti-hepatitis C virus antibody and had high serum concentrations of HBV DNA. |
| 2. Did the non-exposed cohort draw from the same community? | yes | Hospital controls. |
| 3. Ascertainment of exposure? | yes | Medical records. |
| 4. Outcome of interest was not present at start of study? | yes | Curative resection was defined as a complete resection of all macroscopically evident tumors. Absence of tumor cells along the parenchymal transection line was confirmed histologically. No remaining tumor was detected in the remnant liver by computed tomography (dynamic study) 3–4weeks after surgery. |
| 5A. Study controls for age? | yes | Age, gender distribution, the proportion of patients with HBeAg positivity, viral load, the results of laboratory tests, and Child–Pugh classification did not differ between groups. Type of resection, tumor size, tumor number, differentiation of main tumor, prevalence of portal invasion, cancer stage according to UICC classification, 22 severity of active hepatitis as well as degree of hepatic fibrosis in non-cancerous hepatic tissue showed no difference between groups. |
| 5B. Study controls for any additional factor? | yes | Age, gender distribution, the proportion of patients with HBeAg positivity, viral load, the results of laboratory tests, and Child–Pugh classification did not differ between groups. Type of resection, tumor size, tumor number, differentiation of main tumor, prevalence of portal invasion, cancer stage according to UICC classification, 22 severity of active hepatitis as well as degree of hepatic fibrosis in non-cancerous hepatic tissue showed no difference between groups. |
| 6. Assessment of outcome by record linkage? | yes | Serum α-fetoprotein concentrations were measured every three months. Ultrasonography, computed tomography, magnetic resonance imaging, chest radiography, or a combination of these was performed every three months. When tumor recurrence was suspected on the basis of a tumor marker, radiologic studies, or both, angiography or biopsy was performed to obtain a definitive diagnosis. |
| 7. Follow-up long enough? | no | The median follow up from operation until the detection of HCC recurrence or the study endpoint (30 April 2006) in this study was 759 days (34–2053). The median follow up for each group was 1117 days (187–2037) for patients receiving lamivudine and 224 days (34–2053) for the controls. |
| 8. Adequacy of Follow Up of Cohorts? | no | Without description. |

Other characteristics of included studies *[ordered by date of publication]*

|  | **TB (T/C) (**  μmol/L**)** | **ALT (T/C) (** **U/L)** | **AST (T/C) ( U/L)** | **ALB (T/C) (mg/dL)** | **%Child-Pugh class A (T/C)** | | **%Child-Pugh class B (T/C)** | **%** **HBeAg+ (T/C)** |  |
| --- | --- | --- | --- | --- | --- | --- | --- | --- | --- |
| **Kubo 2007** | 13.6/13.6 | 53/56 | 44/40 | 38/37 | | 78.6/80 | 21.4/20 | 78.6/50 | |
| **Kuzuya 2007** | 13.6/15.3 | 56.6/54.2 | NA | 37/37 | | NA | NA | 25/6.1 | |
| **Yoshida 2008** | 25.5/15.3 | 54/36 | NA | 34/38 | | 60.7/71.8 | NA | 24/15 | |
| **Chuma 2009** | 15.3/15.3 | 43.1/37.7 | NA | 40/39 | | 85/90 | 15/10 | 50/43.3 | |
| **Koda 2009** | 25.5/22.1 | 78/54 | 77/56 | 33/35 | | 63.3/80 | 33.3/15 | 36.7/40 | |
| **Chan 2011** | 11.9/11.9 | 58.0/42.5 | 66.5/554.5 | 39/41 | | 100/89.4 | 0/10.6 | NA | |
| **Hann 2011** | NA | NA | NA | 44/42 | | 87.5/80 | 12.5/20 | 37.5/20 | |
| **Wu 2012** | NA | NA | NA | NA | | NA | NA | NA | |
| **Lee 2012** | NA | NA | NA | NA | | NA | NA | NA | |
| **Ke 2013** | 13.5/13.3 | 39/42 | 37/39 | 40.4/40.6 | | NA | NA | 10.6/11.3 | |
| **Nishikawa 2013** | 14.8/15.6 | 52.8/40.0 | 49.8/40.5 | 39.4/41.8 | | NA | NA | 33.8/12.5 | |
| **Su 2013** | 15.3/15.3 | 45/42 | NA | 41/40 | | NA | NA | 15.0/10.2 | |
| **Yin 2013** | NA | NA | NA | NA | | 97.2/97.5 | 2.8/2.5 | 37.2/24.9 | |
| **Yin 2013** | 14.4/15.3 | 47.3/37.5 | 42.1/ 41.5 | 41.5/ 42.2 | | 98.8/100.0 | 1.2/0 | 50.6/31.7 | |

Abbreviations: T, treated; C, control; NA, not available; TB, Total bilirubin; ALT, alanine aminotransferase; AST, aspartate aminotransferase; ALB, serum albumin; HBeAg+, hepatitis B virus e antigen positive;
