# Supplementary material for: Nucleot(s)ide Analogues for Hepatitis B Virus-Related Hepatocellular Carcinoma after Curative Treatment: A Systematic Review and Meta-Analysis
Source: PLoS One. 2014 Jul 24;9(7):e102761. doi: 10.1371/journal.pone.0102761 (PMC4109946; doi:10.1371/journal.pone.0102761)
Supplement: Table S3 — Characteristics of excluded studies. (DOC) [file pone.0102761.s004.doc]

***Supplementary Table S3.*** *Characteristics of excluded studies [ordered by date of publication]*

| **Study** | **Reason for exclusion** |
| --- | --- |
| **Piao, et al (2005)** | Case-control study. It did not meet participant inclusion criteria. Almost half of the participants received transarterial embolization as primary treatment of hepatoma. |
| **Cheng, et al (2006)** | Randomized clinical trial. However, it did not meet the intervention inclusion criteria. It used lamivudine and thymosin alpha 1 as adjuvant therapy. |
| **Kim, et al (2009)** | It did not meet the participant inclusion criteria. Most participants received transarterial chemoembolization as primary treatment of hepatoma. |
| **Li, et al (2010)** | Prospective cohort study. It did not meet participant inclusion criteria. The participants did not receive curative treatment of hepatocellular carcinoma. The mean tumor diameter is 7.1 cm in treatment group and 8.5 cm in control group. Meanwhile, the presence of portal vein tumor thrombus is 30.2% in treatment group and 27.8% in control group. |
| **Inuzuka, et al (2010)** | Just abstract. Data reprised in Nishikawa 2013. |
| **Chan, et al (2010)** | Just abstract. Data reprised in Chan 2011. |
| **Jeong, et al (2011)** | Retrospective study. Just abstract. And it did not identify whether the postoperative antiviral agent is nucleoside analogue or interferon. Communication with the authors is also failed. |
| **Urata, et al (2012)** | A retrospective study. It did not meet participant inclusion criteria. In antiviral therapy group, 46 patients received antiviral therapy before or after the development of HCC. |
| **Huang, et al (2013)** | A retrospective study. It did not meet participant inclusion criteria. The presence of vascular invasion is 7.7% in antiviral group and 5.7% in no antiviral group. And the mean diameter of the tumors is beyond 5.2 cm. |
